# Formal description of Treponema pallidum subsp. pallidum, comb. nov., Treponema pallidum subsp. pertenue, comb. nov., Treponema pallidum subsp. endemicum, subsp. nov. and emended description of Treponema pallidum

**DOI:** 10.1099/ijsem.0.007245

**Published:** 2026-07-23

**Authors:** Steven J. Norris, Diane G. Edmondson, Bridget D. De Lay, Karan G. Kaval, Tsute Chen, Nicole A. P. Lieberman, Alexander L. Greninger, Klára Janečková, David Šmajs

**Affiliations:** 1 Department of Pathology & Laboratory Medicine, UTHealth Houston McGovern Medical School, Houston, TX, USA; 2 Department of Microbiology and Molecular Genetics, UTHealth Houston McGovern Medical School, Houston, TX, USA; 3 Department of Molecular Genetics, The ADA Forsyth Institute, Somerville, MA 02143, USA; 4 Department of Laboratory Medicine and Pathology, University of Washington School of Medicine, Seattle, WA, USA; 5 Department of Biology, Masaryk University, Brno, Czech Republic

**Keywords:** bejel, endemic syphilis, *Treponema pallidum*, syphilis, yaws

## Abstract

This article represents the valid publication of the names of three subspecies of *Treponema pallidum*. These subspecies were first described by Robert M. Smibert in 1984 and correspond with organisms that cause syphilis (subsp. *pallidum*), yaws (subsp. *pertenue*) and bejel (subsp. *endemicum*) in humans. The subspecies nomenclature has been widely used in the literature since that time but has not been validly published due in part to the availability of only limited genetic information. The designation of these subspecies is now supported by a large number of genomic sequences indicating a clear separation of these three closely related yet genetically distinct groups, as demonstrated in this study by phylogenetic analysis of a representative group of strains from each subspecies and the closely related lagomorph pathogen *Treponema paraluiscuniculi*. Subspecies-specific regions were also identified by genomic comparisons to provide an additional means of distinguishing the *T. pallidum* subspecies and *T. paraluiscuniculi* without complete genomic sequencing. Finally, the Gauthier^T^ (=BEI Resources NR-60826^T^=DSM 120346^T^) and Bosnia A^T^ (=BEI Resources NR-60824^T^=DSM 120347^T^) strains are herein designated as the type strains of *T. pallidum* subsp. *pertenue* and *T. pallidum* subsp. *endemicum*, respectively, whereas Nichols^T^ (=BEI Resources NR-59701^T^=DSM 117211^T^) and SS14 (=BEI Resources NR-60825=DSM 120345) represent type and reference strains of *T. pallidum* subsp. *pallidum*, respectively. Overall, this article corroborates the many prior reports that support the formal establishment of the three *T. pallidum* subspecies and provides additional guidelines for their distinction.

## Data Summary

The GenBank/ENA/DDBJ accession numbers for the complete genomes of *Treponema pallidum* subsp. *pallidum* Nichols^T^, *T. pallidum* subsp. *pertenue* Gauthier^T^ and *T. pallidum* subsp. *endemicum* Bosnia A^T^ and reference strain *T. pallidum* subsp. *pallidum* SS14 are CP004010, CP002376, CP007548 and CP004011, respectively, and for the 16S rRNA gene of *T. pallidum* subsp. *pallidum* Nichols^T^ is M88726. Fig. S1, Tables S1 and S2, File S1 and Movie S1 have been deposited in the Figshare repository and are publicly available at https://doi.org/10.6084/m9.figshare.33070370[1] .

## Introduction

Members of a closely related group of host-dependent and morphologically indistinguishable spirochaetes cause syphilis, yaws, bejel (also known as endemic syphilis) and pinta in humans, a yaws-like infection in non-human primates (NHPs) and venereal spirochetosis in rabbits and hares [2–13]. In 1905, Schaudinn and Hoffmann [14] first identified *Spirochaeta pallida* as the organism that causes syphilis; the name was emended to *Treponema pallidum* in recognition of several distinctions from other known *Spirochaeta* [15]. Schaudinn and Hoffmann’s discovery incited a flurry of scientific activity. Within the same year, Castellani [16] determined that the tropical disease yaws was caused by a spirochaete identical in appearance to *T. pallidum*, which was later called *Treponema pertenue*; recent studies have shown that genetically indistinguishable organisms also cause infections in NHPs [12]. Later in the twentieth century, bejel, a disease transmitted during childhood by skin-to-skin contact, was found to be caused by a variant of *T. pallidum*; in addition, pinta, a tropical disease typically causing only skin lesions, is caused by infection with an organism identical in appearance to *T. pallidum* referred to as ‘*Treponema carateum*’ [2]. Finally, *Treponema paraluiscuniculi* (also referred to as ‘*Treponema paraluisleporidarum’*) is associated with sexually transmitted infections in rabbits and hares [8–10, 13]. Properties of these organisms, which can be collectively called the *T. pallidum*-related group or complex, are summarized in Table 1. At present, the phenotypes are defined primarily by infectivity, pathogenesis and cross-immunity patterns in humans and animals, highlighting the need for expanded analysis of the phenotypic characteristics of these organisms.

**Table 1.** Differential characteristics of members of the *T. pallidum*-related group

Characteristic	*T. pallidum* subsp.			‘*T. carateum*’	*T. paraluiscuniculi*
	*pallidum*	*pertenue*	*endemicum*		
***Natural host(s)***					
Humans	+	+	+	+	−
Non-human primates	−	+	−	−	−
Rabbits or hares	−	−	−	−	+
***Nature of infection in natural host***					
Systemic; may affect most internal organs, causing neurologic and cardiovascular manifestations	+	−	−	−	−
Lesions usually restricted to skin, oral cavity, bone or cartilage	−	+	+	+	+
Sexually transmitted	+	-(+)*	+^†^	−	+
Typically transmitted by skin-to-skin contact, often during childhood	−	+	+	+	
***Geographical distribution***					
Worldwide	+	−	−	−	+
Found in tropical environments in many areas of the world	−	+	−	−	−
Found in tropical countries in Central and South America	−	−	−	+^‡^	−
Usually restricted to semi-arid or temperate regions in the Middle East, Africa and Southeast Asia; increasing evidence of worldwide spread through sexual transmission	−	−	+	−	−
***Cutaneous lesions can be produced by experimental infection of***					
Rabbits	+	+	+	−	+
Hamsters	+^§^	+^§^	+^§^	−	
Mice	−	−	−	−	
Guinea pigs	+^¶^	+**	−	−	
Non-human primates	+	+	+	+	

*Sexual transmission likely occurs in non-human primates.

†An increasing number of cases of sexual transmission in humans have been demonstrated.

‡No cases of *T. carateum* infection have been reported in the past 50 years.

§Hamsters are less susceptible to subsp. *pallidum* than to subsp. *pertenue* or *endemicum*.

¶Lesions are only slightly indurated and do not ulcerate [70].

**Guinea pigs have less frequent and severe lesions following inoculation with subsp. *pertenue* as compared to subsp. *pallidum*. Lesion development with subsp. *pertenue* is more frequent in young guinea pigs [114].

**Fig. 1.**
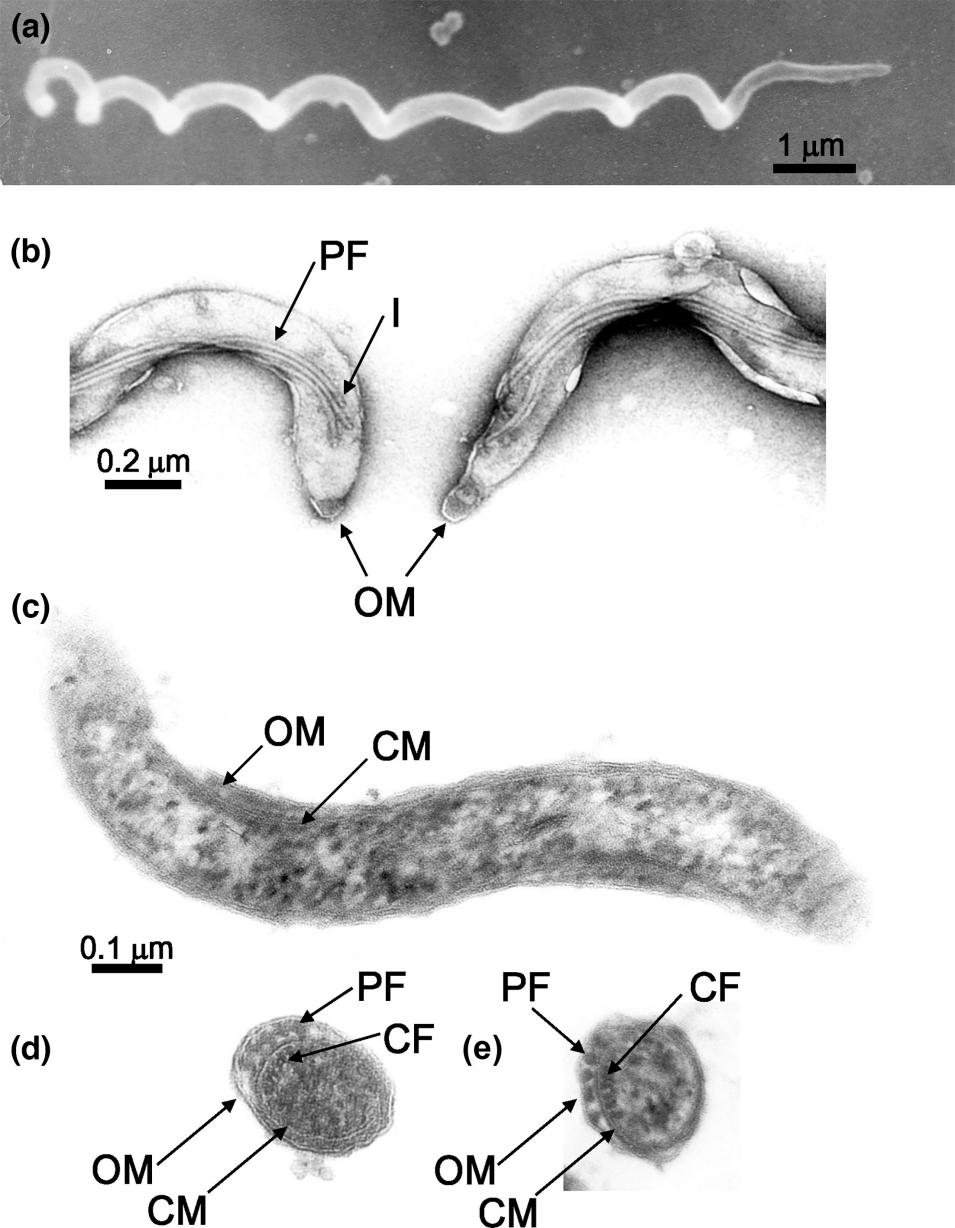
Cellular morphology of *T. pallidum*. *T. pallidum* subsp. *pallidum* Nichols^T^ is shown and is representative of the other subspecies. (a) Scanning electron micrograph, showing the coiled structure of the bacterium. (b) Transmission electron micrograph of the ends of two intact organisms negatively stained with 1% uranyl acetate. Periplasmic flagella (PF), their flagellar motor insertion points (I) and an outpouching of the outer membrane (OM) at the cell tips are indicated. (c–e) Longitudinal and cross-sections of *T. pallidum* fixed with 1% paraformaldehyde, post-fixed with 1% uranyl acetate and processed for electron microscopy with ultrathin sectioning and uranyl acetate and lead citrate staining. Cytoplasmic filaments (CF) lie just underneath the cytoplasmic membrane (CM) and run the length of the organism in parallel with the periplasmic flagella.

These organisms share a slender, spiral-shaped morphology (Fig. 1) characterized by a diderm membrane structure, an outer membrane that lacks lipopolysaccharide, and the presence of both periplasmic flagella and cytoplasmic filaments [17–22]. They have a characteristic motility consisting of rotation, flexion and reversal activities (Movie S1 in [1], available in the online Supplementary Material). Until 1981, *T. pallidum* could only be propagated by inoculation of rabbits or other laboratory animals [23]. At that time, Fieldsteel *et al*. introduced a system utilizing Sf1Ep rabbit epithelial cells, a specialized tissue culture medium (TpCM1) containing Eagle’s MEM, foetal bovine serum and other additives, and incubation in an atmosphere with 1.5% O_2_ and 5% CO_2_ that allowed limited multiplication of *T. pallidum* for up to 21 days [17, 24, 25]. In 2018, replacement of Eagle’s MEM with CMRL 1066 in the medium (TpCM2) of this system was found to promote continuous culture of *T. pallidum* [26–28]. In this system, *T. pallidum* grows with a characteristically long optimal generation time (29 to 44 h), depending on the strain [29, 30]; it also involves adherence to, and the apparent acquisition of nutrients from, the mammalian cell monolayer. Several syphilis and endemic syphilis/bejel isolates have been propagated continuously *in vitro* [26, 30]. Long-term survival and growth of some yaws isolates has also been achieved (J. Bosák and D. Šmajs, unpublished data; D. G. Edmondson and B. D. De Lay, unpublished data), but thus far *in vitro* culture of *T. paraluiscuniculi* strains has not been reported. It was demonstrated recently that human foreskin fibroblast cultures, as well as certain human carcinoma and transformed trophoblast cell lines, can effectively replace Sf1Ep cells in this culture system [31–33]. The *in vitro* culture system has enabled the direct cultivation of strains from patient specimens [34], as well as the isolation of clonal populations [35]. The availability of *T. pallidum* culture has accelerated research involving these fastidious organisms, including studies of their growth characteristics and requirements [30, 35], gene transcription [36–38], proteomics [38–40], antimicrobial susceptibility [41–47], protective antigen candidates [48] and genetic manipulation [47, 49–53].

Prior to 1984, *T. pallidum* and *T. pertenue* were considered separate species, with no named subspecies. In the edition of *Bergey’s Manual of Systematic Bacteriology* published that year, Dr. Robert M. Smibert introduced three subspecies of *T. pallidum: T. pallidum* subsp. *pallidum*, *T. pallidum* subsp. *pertenue* and *T. pallidum* subsp. *endemicum* [54]. This change in nomenclature was based on the DNA–DNA saturation reassociation kinetics studies of Miao and Fieldsteel [55], who found that DNA preparations from *T. pallidum* and *T. pertenue* strains cross-hybridized and were indistinguishable by this method; from this finding, Dr Smibert reasoned that the designation of these taxa as two separate species was no longer justified. The *T. pallidum* subsp. *pallidum*, *T. pallidum* subsp. *pertenue* and *T. pallidum* subsp. *endemicum* subspecies designations were based on their correspondence to the diseases venereal syphilis, yaws and bejel (endemic syphilis), respectively. Although initially based solely on disease causation phenotypes, these subdivisions are now well supported by complete or near-complete genomic sequences obtained by high-throughput sequencing of over 3,000 *T*. *pallidum* isolates and clinical specimens. The proposed subspecies are very closely related genetically, with over 99.7% overall sequence identity [56]. Prior to the availability of genomic sequences, it had been proposed that syphilis, yaws and bejel were all caused by a single organism and that the differences in symptomology and disease transmission were due to environmental factors, such as varied climates [57, 58]. However, numerous phylogenetic analyses have demonstrated that three genetically distinct groups of *T. pallidum* organisms are predominantly associated with syphilis, yaws (and treponematosis in NHPs) and bejel [56, 59–65]. The three subspecies designations have thus withstood the test of time and scientific scrutiny and have been utilized in hundreds of publications since their introduction over 40 years ago.

To date, the nomenclature corresponding to the *T. pallidum* subspecies has not been considered to be validly published because of issues regarding compliance with rules within the International Code of Nomenclature of Prokaryotes [66]. Specifically, genomic information supporting the designation of subspecies had been sparse until the last decade, and type strains had not been established or made available at two repositories. The purpose of this article is to describe the subspecies, validly publish their names, report the deposition of representative type strains and provide additional guidelines for differentiating the subspecies through the use of subspecies-specific regions (SSRs).

## Methodology

### *T. pallidum* strains

*T. pallidum* subsp. *pallidum* Nichols^T^ was initially isolated from the cerebrospinal fluid of a neurosyphilis patient in 1912 by Nichols and Hough [67] and was obtained from Dr James N. Miller at the David Geffen School of Medicine at the University of California at Los Angeles by S.J.N. in 1982. Isolated in 1977 in Atlanta, GA, USA, in association with the US Centers for Disease Control and Prevention (CDC), *T. pallidum* subsp. *pallidum* SS14 was from a secondary syphilis lesion of a patient following an erythromycin treatment failure [68]. *T. pallidum* subsp. *pertenue* Gauthier^T^ was isolated by Gastinel *et al.* in 1960 [69] through the inoculation of a hamster with skin lesion exudate from a human patient with yaws in the Congo. *T. pallidum* subsp. *endemicum* Bosnia A^T^ was obtained by E. I. Grin in 1950 using the inoculation of hamsters with exudate from an oral lesion from a patient with bejel, as later described by Turner and Hollander [70]. The SS14, Gauthier^T^ and Bosnia A^T^ strains were acquired as frozen stocks by S.J.N. from David L. Cox at the CDC in 1997. All of these strains had been maintained long-term by serial passage in laboratory animals (primarily rabbits), and freezing was introduced as a means of decreasing the need for serial passage beginning in 1975. Frozen stocks of the four strains for deposition at BEI Resources in Manassas, VA, USA, and at the Deutsche Sammlung von Mikroorganismen und Zellkulturen (DSMZ) in Braunschweig, Germany, were prepared by intratesticular inoculation of rabbits [23], extraction in TpCM-2 medium [26, 28], centrifugation to remove tissue debris, the addition of 10% (v/v) glycerol and freezing at −80 °C [26, 28].

### Genomic sequence data

Complete or near-complete consensus genomic sequences for analysis in this study were obtained from the National Center for Biomedical Informatics (NCBI) website (https://www.ncbi.nlm.nih.gov/). In addition, a library of genome sequence .fastq files and associated information from 3,008 *T*. *pallidum* specimens collected worldwide was assembled by N.A.P.L. and A.L.G. This collection was utilized to analyse proposed SSRs for the consistency of the sequences within each of the subspecies.

### Phylogenetic analysis

To generate a maximum-likelihood phylogeny, synthetic reads for the 81 strains shown in Table S1 (in [1]) were generated and assembled to the SS14 reference to a depth of ~50 x and variants called using bcftools v1.21 [71, 72]. Regions of the genome known to undergo positive selection and/or recombination [60, 61, 65] were masked, and a single nucleotide variation (SNV)-only phylogeny was generated using IQ-TREE v2.0.3 [73].

### Subspecies-specific regions

To identify regions potentially useful for differentiating the *T. pallidum* subspecies, the genome sequences of 11 representative strains [*T. pallidum* subsp. *pallidum* Nichols^T^, SS14; *T. pallidum* subsp. *pertenue* Gauthier^T^, CDC2, 6RUM2090716, 22LMF5290815; *T. pallidum* subsp. *endemicum* Bosnia A^T^, Iraq B, and C279; *T. paraluiscuniculi* Cuniculi A and V3603-13 (L2)] were selected, along with 3 *T. pallidum* subsp. *pallidum* Nichols^T^ clones (TpN-CL1, TpN-CL-3 and TpN-CL8) to provide a measure of the effects of intrastrain heterogeneity [35, 74, 75] (Fig. S1 in [1]). The genome sequences were aligned using MAFFT [74, 75], and DNASTAR MegAlign Pro was used to identify sequence variations and to aid in the manual optimization of the alignment by repositioning relative indels or N regions without altering the nucleotide sequences. The aligned sequences were then evaluated for regions that were identical among members within each subspecies but differed between subspecies. Twenty-eight potential SSRs that had a >2 nt difference between subspecies were then each compared to the *Treponema* consensus-assembled genomes available in the NCBI database using blastn, using nucleotide segments containing the candidate SSR flanked by 100 nt of additional sequence on either side. This approach further reduced the number of SSR candidates to 8.

To further examine the consistency of the SSRs within each of the *T. pallidum* subspecies, each of the 8 candidate subspecies-specific regions were evaluated in a panel of 3,008 paired-end sequencing read sets from *T. pallidum* collected from human and NHP subjects; this database was assembled as part of a project to create a centralized, easily accessible online resource that encompasses all publicly available *T. pallidum* genome sequences and sequencing read sets (N. A. P. L. and A. L. G., unpublished data). Raw paired-end sequencing reads were downloaded from the European Nucleotide Archive (ENA), host reads were removed using kraken2 v2.1.3 [76] and quality and adapter trimming was performed using trimmomatic v0.39 [77]. For strains with only consensus GenBank entries, synthetic paired reads were generated using InSilicoSeq v2.0.1 [78] with a Novaseq 6000 error profile. Processed or synthetic reads were assembled to the subspecies *pallidum* SS14 reference (CP004011) or the subspecies *pertenue* SamoaD reference (CP002374) using Bowtie2 v2.5.2 [79] with default settings and deduplicated with Picard v2.26.3 [80]. SSR region consensus sequences were called directly from the genome assemblies, including flanking regions, using samtools consensus v1.21 [71, 72], requiring a depth of at least five reads and an allele frequency of 0.8. SSR consensus sequences with ambiguities due to low coverage were excluded from the analysis. The data from this analysis are included in File S1 [1]; data points excluded because of low coverage or sequence ambiguity are marked as not available (‘NA’). Although sequence data for 3,008 specimens were available at the time, only those with sufficient read coverage (designated with phylo_y_pos numbers in File S1 in [1]) were included in the analysis of SSR consistency; duplicate read sets from the same strain or specimen were also excluded, bringing the number analysed to 2,556. ssrE was assessed only for the absence or presence of the ~379 bp relative deletion and not for sequence heterogeneity that exists in that region. The SSRs for *T. pallidum* subsp. *endemicum* were assessed using genome sequences available on NCBI because of low read coverage in many of the SSRs in the few available read sets.

## Results and discussion

### Type and reference strains

Properties of the type and reference strains described in this article are provided in Table 2. The Nichols^T^ strain was recently designated as the type strain of *T. pallidum* [81], and in accordance with Rule 13d of the International Code of Nomenclature of Bacteria, it is also the type for the subspecies bearing the same name as the species. Street Strain 14 (SS14) is included as a reference strain for *T. pallidum* subsp. *pallidum* because it is representative of a prevalent subset of closely related strains often called the SS14 cluster, clade or lineage [82]. The Gauthier^T^ strain is the type strain of *T. pallidum* subsp. *pertenue* and was isolated from a yaws patient in an endemic region in the Congo. The *T. pallidum* subsp. *endemicum* type strain Bosnia A^T^ was obtained in 1950 by Dr E. I. Grin from a bejel patient in Bosnia, where at the time the disease had a prevalence as high as 60% in some villages [83, 84]. While yaws and bejel were once prevalent in many endemic areas, their incidence has decreased dramatically as a result of improved living conditions and eradication programmes [2, 84]. The two *T. pallidum* subsp. *pallidum* strains were isolated by rabbit inoculation, while Gauthier^T^ and Bosnia A^T^ strains were first inoculated into hamsters and later transferred to rabbits. All available *T. pallidum* strains have been isolated by animal inoculation, with the exception of a few strains that have been isolated recently by direct inoculation of *in vitro* cultures [34]. Frozen stocks of the three type strains and one reference strain have been deposited at BEI Resources and the DSMZ with the accession numbers provided in Table 2.

**Table 2.** Properties of *T. pallidum* type and reference strains

Subspecies	Strain	Isolated	Source	Isolation location	Reference	NCBI genome accession	BEI accession	DSMZ accession
*T. pallidum* subsp. *pallidum*	Nichols^T^ (type)	1912	Cerebrospinal fluid of neurosyphilis patient in relapse following Salvarsan treatment	Washington, DC, USA	[67]	CP004010.2	NR-59701^T^	DSM 117211^T^
*T. pallidum* subsp. *pallidum*	SS14 (reference)	1977	Skin lesion exudate from a patient with secondary syphilis following erythromycin treatment failure	Atlanta, GA, USA	[68]	CP004011.1	NR-60825	DSM 120345
*T. pallidum* subsp. *pertenue*	Gauthier^T^ (type)	1960	Skin lesion exudate from patient with yaws	Congo	[69]	CP002376.1	NR-60826^T^	DSM 120346^T^
*T. pallidum* subsp. *endemicum*	Bosnia A^T^ (type)	1950	Exudate from oral ‘mucous patch’ lesions in a male patient with bejel (endemic syphilis)	Bosnia	[70]	CP007548.1	NR-60824^T^	DSM 120347^T^

### Phylogenetic comparisons

As mentioned previously, many prior phylogenetic studies have indicated the clear delineation of the three subspecies [56, 59–65]. For the analyses performed for this article, we selected genomic sequences from *T. pallidum* subspecies and *T. paraluiscuniculi* that were (1) available as assembled genomes on the National Center for Biomedical Informatics website (https://www.ncbi.nlm.nih.gov/), (2) >95% complete (i.e. contained <5% ‘Ns’) and (3) representative of different geographic locations (Table S1 in [1]). For *T. pallidum* subsp. *pallidum*, 47 representative sequences were selected from a much larger number of available sequences. For *T. pallidum* subsp. *pertenue*, 9 complete or near-complete sequences from humans and 15 from NHPs were selected, and 8 available genome sequences of *T. pallidum* subsp. *endemicum* were utilized. Two complete *T. paraluiscuniculi* sequences available in nucleotide databases were included for comparison. It should be noted that only 22 of the genomic sequences utilized in this study were from bacterial isolates (all initially propagated by animal inoculation). The remaining 59 sequences (and nearly all of the additional genome sequences available on NCBI or ENA) were obtained using DNA purified directly from human or NHP subject specimens. No genetic information or isolates are available for ‘*Treponema carateum*’, so it was excluded from this analysis. The recent development of a procedure for direct *in vitro* culture of *T. pallidum* strains [34] promises to greatly increase the number of isolates available for study.

**Fig. 2.**
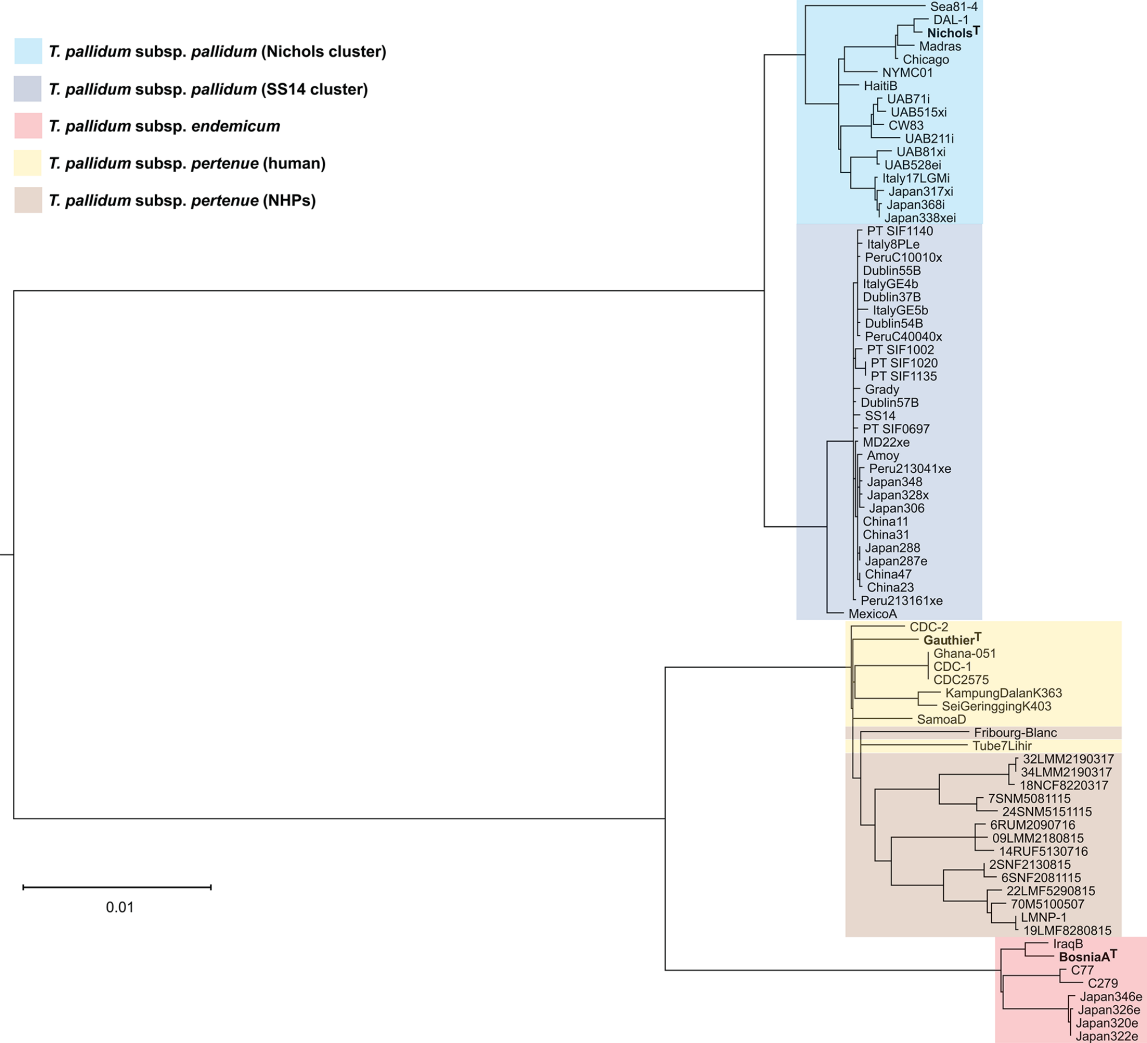
Phylogenomic tree derived from a maximum-likelihood analysis of genome sequences representative of the three *T. pallidum* subspecies (Table S1 in [1]). Colour coding corresponds to each taxon. In addition, the Nichols- and SS14-associated clusters of *T. pallidum* subsp. *pallidum* are coloured differentially, as are the *T. pallidum* subsp. *pertenue* genomic sequences for specimens obtained from humans or non-human primates. *T. paraluiscuniculi* specimens were not included in this figure to permit better visualization of the branching patterns within the *T. pallidum* group.

The results of the maximum-likelihood analysis of the *T. pallidum* sequences (Fig. 2) illustrate the clear separation of the *T. pallidum* subspecies demonstrated in prior studies. Also evident is the existence of two subsets of *T. pallidum* subsp. *pallidum* genomic sequences representing the Nichols-related cluster and the SS14-related cluster as described previously [56, 59, 61, 64, 65]. *T. pallidum* subsp. *pertenue* forms a cohesive branch in the phylogenetic tree [12]; however, the human- and NHP-derived specimens generally tend to cluster separately within the subspecies. Although relatively few specimens representative of *T. pallidum* subsp. *endemicum* are available, this group is well-separated from the other subspecies. Historically, *T. pallidum* subsp. *endemicum* was thought to be predominantly transmitted by skin-to-skin contact or through contaminated fomites (such as drinking vessels). In recent years, there is increasing evidence of sexual transmission of *T. pallidum* subsp. *endemicum* in humans, as indicated by sexually transmitted infections in Cuba (exemplified by specimens C77 and C279) [85–87] and in four cases identified in Japan [88–91] (Fig. 2).

### Identification of subspecies-specific regions

To provide additional information to aid in the differentiation of *T. pallidum* subspecies, we sought to identify genomic sequences that are specific for each of the subspecies. To begin this process, the genome sequences of nine *T*. *pallidum* strains representative of the three subspecies, three Nichols^T^ strain clones and two *T. paraluiscuniculi* strains were selected. These genome sequences were aligned and then examined for regions that were consistently conserved within the strains of one or more of the subspecies, but different from other subspecies (Fig. S1 in [1]); a secondary consideration was whether the *T. paraluiscuniculi* sequences also had a unique ‘signature’. Regions were selected that contained relative indels of two or more base pairs, multiple SNVs or combinations of the two; regions containing tandem repeats, homopolymeric stretches and sequences exhibiting identity with other regions of the genome were excluded. Each of the 28 candidate regions initially selected was then subjected to blastn searches against the NCBI databases to determine whether the observed sequence differences within a candidate signature region were consistently conserved within each subspecies. This process resulted in the selection of eight SSRs, which were designated ssrA through ssrH (Table S2 in [1]).

To further examine the consistency of the SSR sequences within each subspecies, each SSR was analysed for sequence identity against a compendium of 2556 sequence read sets downloaded from the ENA that represented *T. pallidum* specimens collected worldwide by many different research groups (File S1 in [1]). This panel encompassed specimens previously identified as containing *T. pallidum* subsp. *pallidum* (*N*=2184), subsp. *pertenue* (*N*=362) and subsp. *endemicum* (*N*=10). The actual number of specimens analysed for each SSR was less than these numbers, because some of these results did not meet the requirements for read depth and the lack of sequence ambiguity. For each SSR, variants were numbered 1, 2 or 3 based on whether the variant predominantly corresponded to *T. pallidum* subsp. *pallidum*, subsp. *pertenue* or subsp. *endemicum*, respectively. If more than one variant was observed in a subspecies, each of these variants was given a number and letter designation, such as 1a, 1b and 1c, with 1a having the highest prevalence.

**Fig. 3.**
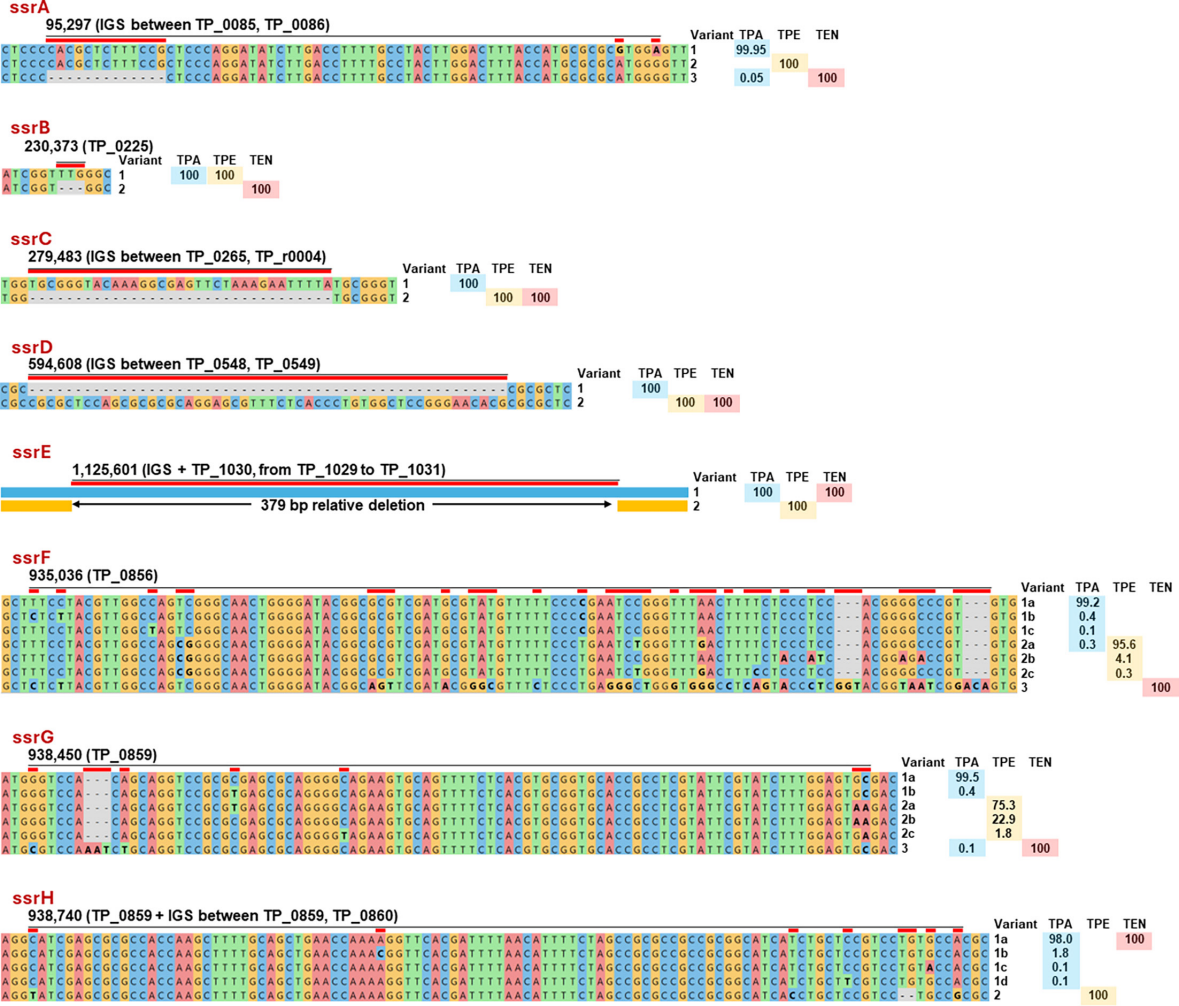
SSRs identified through comparison of strains of *T. pallidum* subsp. *pallidum* (here abbreviated TPA), *T. pallidum* subsp. *pertenue* (TPE) and *T. pallidum* subsp. *endemicum* (TEN). The regions are designated ssrA through ssrH, and the beginning coordinate in the *T. pallidum* subsp. *pallidum* Nichols^T^ genome (CP004010.2) and the associated genes or intergenic sequences (IGSs) are indicated for each SSR. The corresponding coordinates for the SSRs in the three type strains and *T. paraluiscuniculi* Cuniculi A, as well as additional information, are provided in Table S2 in [1]. The variants found in a database of genomic data from 2,556 *T*. *pallidum* specimens (including 2,184 subsp. *pallidum*, 362 subsp. *pertenue* and 10 subsp. *endemicum* specimens) are indicated; the variant numbers (1, 2 or 3) correspond to those of each respective subspecies, whereas letters (e.g. 1a and 1b) denote variants that were observed within that subspecies. The percentage of each variant observed in the three subspecies is indicated to the right of each SSR alignment. ssrE was characterized solely by the presence or absence of a large (~379 bp) deletion, in that SNVs and other genetic variations are common in the area of the relative insertion. ssrA through ssrE, which are primarily indel-based and provided highly consistent results within each subspecies, whereas ssrF through ssrH, which are mostly SNV-based, exhibited a higher frequency of intra-subspecies variants.

The variants observed in each SSR and their corresponding percentage in each subspecies are indicated in Fig. 3 and in File S1, Tables 2 and 3 (in [1]). The analysis revealed that ssrA through ssrE, which are primarily based on the presence or absence of indels (ranging in length from 2 to 379 bp), yielded highly consistent results within each subspecies. In all cases except ssrA, there was 100% concordance between the subspecies of the specimen and the variant detected; in ssrA, only 1 of 2,058 *T*. *pallidum* subsp. *pallidum* specimens differed from the canonical variant 1 genotype, with the single exception having the same sequence as *T. pallidum* subsp. *endemicum* (indicating a likely recombination event in that location). These results indicate that ssrA through ssrE could be used to reliably determine the subspecies of a *T. pallidum* strain (using, for example, site-specific PCR and sequencing) without requiring complete genome sequencing.

ssrF, ssrG and ssrH are primarily based on SNV differences; these regions exhibited a higher number of variants and thus a more complex pattern of concordance (Fig. 3). For example, the *T. pallidum* subsp. *pallidum* specimens had four variants in ssrF with frequencies of 2,207 (1a), 9 (1b), 2 (1c) and 6 (2a) specimens, with the latter being identical to the predominant *T. pallidum* subsp. *pertenue* genotype. The most interesting of these variances is in ssrG of *T. pallidum* subsp. *pertenue*, in which 77 of 336 specimens (22.9%) are of variant 1b, with a single base pair difference from the most frequent variant 1 a (75.3%). All of the specimens with this ssrG-1b genotype are from sub-Saharan Africa, with 61 being from human subjects and 17 from NHP subjects. Such SSR variations may be useful as markers of different genotypes within a subspecies. While ssrF, ssrG and ssrH may be useful in distinguishing the *T. pallidum* subspecies, the results obtained may not be as definitive as for the indel-based SSRs.

**Fig. 4.**
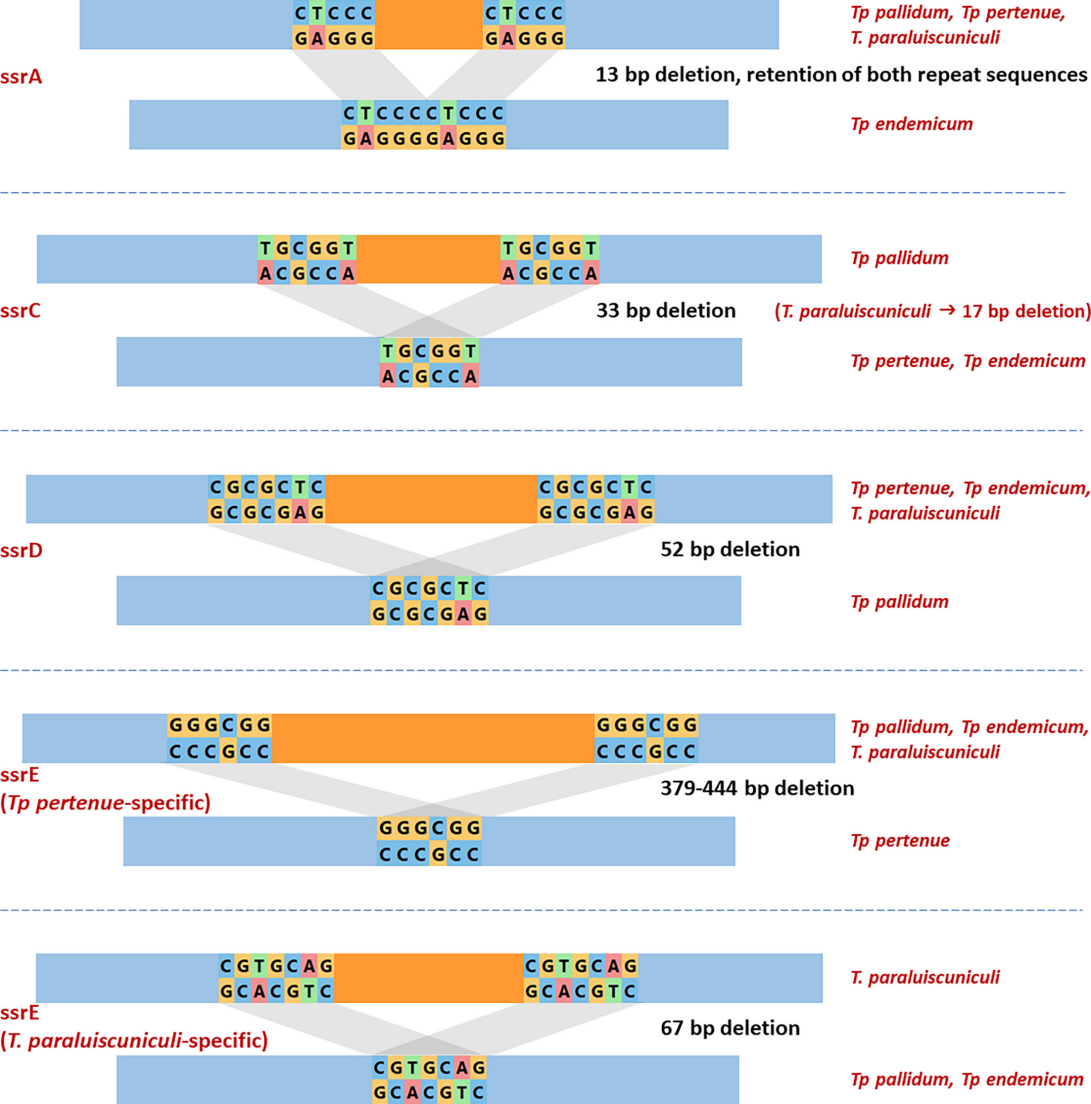
Short direct repeats are involved in subspecies-specific insertion–deletions. At the five SSRs shown, the *T. pallidum* subspecies or *T. paraluiscuniculi* are differentiated by the presence or absence of deletions ranging in size from 13 bp (ssrA) to ~379 bp (ssrE). In each case, identical sequences with lengths from 5 to 7 bp flank the region that undergoes the deletion. In the taxa in which the deletion has occurred (shown on the right side of the diagram), an apparent crossover event between the two repeated sequences resulted in the deletion of the intervening sequence, resulting in a single copy of the repeat sequence. ssrA differs in this regard, in that both copies of the repeat sequence are retained. In ssrC, *T. paraluiscuniculi* has a unique, 17 bp deletion that does not have corresponding flanking direct repeats in *T. pallidum* subsp. *pallidum*. In ssrE, the large deletion in *T. pallidum* subsp. *pertenue* encompasses the region in which *T. pallidum* subsp. *pallidum* and *T. pallidum* subsp. *endemicum* have a 67-bp deletion relative to *T. paraluiscuniculi*. The lengths of the deleted regions are not shown to scale.

A surprising finding was that the relative insert regions in ssrC, ssrD and ssrE were flanked by short, identical sequences (consisting of 5 to 7 bp) on either end; a similar arrangement was observed in a 67 bp relative insertion in *T. paraluiscuniculi* in the ssrE region (Fig. 4, Table S2 in [1]). In the taxa having the relative deletion, the ‘insert’ was lost and only one of two flanking sequences was present. (ssrA represents an unusual case, in which both flanking repeat sequences are retained in the relative deletion (Fig. 4).) The most logical explanation is that, in each locus, a crossover recombination event occurred between the two flanking sequences, resulting in the deletion of the intervening sequence. Similar recombinations have been observed previously at other sites in *T. pallidum* subsp. *pallidum* Nichols^T^ clones [35]. This type of event would be expected to be unidirectional, with the deletion of the intervening region being irreversible (except in the conceivable instance of horizontal transfer from other organisms). These events thus provide examples of genome reduction, as has been noted in prior studies [92]. All of the deletion-related SSRs are located in predicted intergenic sequences, except ssrE, which results in a change in the 5′ end of the gene encoding the *T. pallidum* repeat protein TprL (Table S2 in [1]). The ssrE-related deletion observed in *T. pallidum* subsp. *pertenue* strains results in decreased expression levels of TprL and a corresponding reduction in antibody responses to TprL in experimentally infected rabbits [93]; it is also predicted to change the N-terminal sequence and part of the *β*-barrel structure of TprL (Table S2 in [1]). The fact that the relative insertion–deletion status is conserved within each of the *T. pallidum* subspecies and *T. paraluiscuniculi* indicates that negative selection is active in some of the taxa, resulting in elimination of organisms in which this deletion has occurred.

A listing of intra-subspecies SSR variants is provided in File S1, Table 8 in [1]. In some cases, these intra-subspecies variations appear to identify subgroups present in a certain geographic area or population. For example, the *T. pallidum* subsp. *pertenue* variant ssrG-2b (representing a single SNV) was consistently found in 61 human and 17 NHP specimens in Africa, differentiating this group from specimens obtained in the Oceania region (Papua New Guinea and Solomon Islands). ssrF-2b, consisting of 5 SNVs relative to the predominant *T. pallidum* subsp. *pertenue* type ssrF2a, was found in a subset of 14 Papua New Guinea specimens from human yaws patients. Of greatest interest is a group of syphilis specimens from diverse locations (Australia, Japan and UK) that contained the ssrF-2a sequence, which is identical to the predominant *T. pallidum* subsp. *pertenue* ssrF type and differs by 5 SNVs from the predominant *T. pallidum* subsp. *pallidum* ssrF-1a sequence; this group had the predominant *T. pallidum* subsp. *pallidum* sequence at all the other SSR sites. These results indicate that the identification of SSR intra-subspecies variants may potentially be of value in identifying subgroups and tracing outbreaks.

An interesting feature of ssrF is a 68-bp sequence in *T. pallidum* subsp. *endemicum* strains (represented by nt 933,050 to 933,117 in the Bosnia A^T^ genome, CP007548.1) that corresponds exactly with the *T. paraluiscuniculi* sequence in that region (Fig. S1 in [1]); this finding has been reported previously [94]. One possibility is that this region of the *T. pallidum* subsp. *endemicum* sequence was replaced by the corresponding sequence in *T. paraluiscuniculi* (or another unidentified or extinct *Treponema* taxon) through a horizontal transfer event. The region is in the middle of the coding sequence for TP_0856, a predicted homologue of FadL (a long-chain fatty acid transport protein), and is predicted to encode some of the extracellular loops; thus, the sequence replacement would likely affect protein function.

The most robust way to differentiate *T. pallidum* subspecies and *T. paraluiscuniculi* would be comparison of the genome-wide SNV profile, requiring whole-genome sequencing, which might be difficult in samples containing a low amount of treponemal DNA or impossible in low-budget settings. An additional approach includes the use of schemes for typing of clinical isolates of syphilis and yaws [85, 95–97] that test loci which are different among isolates of the same subspecies. While this article was in preparation, Pla-Díaz *et al*. [98] published an extensive study on the development of a set of multi-locus sequence typing (MLST) loci for the differentiation of the *T. pallidum* subspecies, as well as detecting additional heterogeneities useful in epidemiological studies. In their analysis, seven regions were identified that can be amplified and sequenced to identify the subspecies of a given strain; a strategy was also included to detect the single-nucleotide changes in the 23S rRNA genes that are associated with macrolide resistance. The goal of the similar analysis described in our article was to identify a set of sequences that were identical among the strains of each subspecies for which genome sequences are available, whereas Pla-Diaz *et al*. [98] included regions that were heterogeneous within subspecies. As a result, some target regions described in the two analyses overlap, while others are different. Limitations of both studies are that (1) only currently available *T. pallidum* strains with near-complete genome sequence coverage could be analysed, with relatively few available in *T. pallidum* subsp. *pertenue*, *T. pallidum* subsp. *endemicum* and *T. paraluiscuniculi*, and (2) *T. pallidum* strains and subspecies will continue to evolve through recombination events, indels and point mutations, so identification criteria may have to evolve with them. Regardless, the two approaches are complementary and will be of value in future studies regarding the biology, pathogenesis and epidemiology of these organisms. In a recent study, Beale *et al*. [99] reported a MinION-based approach called PhyloPlex in which 52 regions containing SNVs were used to differentiate lineages and sublineages within the *T. pallidum* complex, providing a granular analysis of evolutionary relationships that does not require whole-genome sequencing [99].

Advances in the sequencing of ancient DNA have led to the genomic sequencing of several *T. pallidum* samples from human bones ranging in age from an estimated 165 to 5,500 years [64, 98, 100–103]. Thirteen of these sequences were included in the database examined for SSRs (File S1, Table 9 in [1]). Many of these lacked sufficient DNA coverage to provide a meaningful analysis of SSR content, but five of the specimens yielded results for four or more of the eight SSRs. The SSR profiles of specimens AGU007 and SJN003 from Lithuania and Mexico were consistent with previous maximum-likelihood analyses [64, 100], indicating the presence of *T. pallidum* subsp. *pertenue* strains in these regions during the estimated time range of 1463–1632 CE and 1436–1472 CE, respectively. Similarly, the SSR profile of 94A from Mexico (1600–1861 CE) was concordant with prior results indicating that this specimen represents *T. pallidum* subsp. *pallidum* or a closely related ‘sister group’ to this subspecies [64, 98, 100]. In contrast, specimens ZH1540 and GAP009 from Brazil (346–573 CE) and Chile (1250–1485), respectively, contained a mixture of DNA regions corresponding to those found in the modern *T. pallidum* subspecies by both SSR and whole-genome analyses. The oldest specimen examined thus far [101], a 5,500-year-old tibia from Colombia, exhibited the greatest genome sequence divergence from the *T. pallidum* subspecies thus far; this specimen was not included in our SSR analysis. Overall, these observations are consistent with the existence of considerable divergence of *T. pallidum* strains followed by a period of positive selection of more narrowly related genotypes, resulting in the current subspecies [60, 64, 98, 100–103]. They also indicate that examination of the SSRs of ancient specimens (e.g. by PCR) may provide useful information in advance of the more challenging whole-genome analysis.

In conclusion, clear genetic evidence now exists supporting the formal designation of the three subspecies of *T. pallidum*. There is also abundant evidence for the existence of subgroups within each of these subspecies, as would be expected given the continuing evolution and divergence of these closely related organisms. However, it seems unlikely that sufficient information supporting the formation of additional subspecies will accumulate in the future. It could be argued that *T. paraluiscuniculi* should also be designated as a *T. pallidum* subspecies, given their overall genome sequence identity of ~99.2%. In our opinion, more genomic sequence information and phenotypic data are needed to clarify the taxonomic relationship between these rabbit and hare infectious agents and *T. pallidum*. Finally, an improved definition of phenotypic differences of the *T. pallidum* subspecies, other than the sometimes-overlapping transmission patterns, geographic distribution, invasive properties and disease manifestations, is needed. Isolation and characterization of new strains through *in vitro* culture may aid in that endeavour.

## Emended description of *Treponema pallidum* [81]

*Treponema pallidum* (pal’li.dum. L. neut. adj. *pallidum*, pale, pallid).

Basonym: *Spirochaeta pallida* Schaudinn and Hoffmann [14], *Treponema pallida* Schaudinn [15], *Treponema pallidum* (Approved Lists 1980).

Cells are tightly coiled spirochetes ~0.18 µm in diameter by 6–20 µm in length. The wavelength of coils is 1.1 µm, and the amplitude is 0.2–0.3 µm. The ends of the cells are pointed, and a protrusion of the outer membrane at the end is often visible in well-preserved specimens by electron microscopy with negative staining. Two to four periplasmic flagella are inserted into each end of the cell and overlap in the middle of the cell. Motile with rotational and flexing movements. The three subspecies and the closely related species *T. paraluiscuniculi* are all morphologically indistinguishable. Microaerophilic, with an optimal O_2_ concentration in the range of 1–5%.

Continuous culture of *T. pallidum* subsp. *pallidum* and *T. pallidum* subsp. *endemicum* strains has been achieved using co-culture with cottontail rabbit epithelium cells (Sf1EP) in a specialized culture medium at 34 °C in an atmosphere of 1.5% O_2_, 5% CO_2_ and balance N_2_ [26–28, 30]. *T. pallidum* subsp. *pertenue* strains can also be propagated *in vitro* using the same methodology, but multiply at a much slower rate (J. Bosák and D. Šmajs, unpublished data; D. G. Edmondson and B. D. De Lay, unpublished data). *T. pallidum* strains can also be propagated by inoculation of rabbits or hamsters [23, 70].

Obligate pathogens of humans; *T. pallidum* subsp. *pertenue* also causes natural infections in non-human primates. Can cause experimental infection and skin lesions in rabbits, guinea pigs, hamsters and primates. Genomic sequence comparisons indicate a high overall sequence identity of 99.7%, with relatively minor genetic differences defining three subspecies of *T. pallidum* [56]. The subspecies are associated with distinctive clinical symptoms in humans and different patterns of infection in laboratory animals and can be distinguished using MLST or other DNA sequence-based approaches [85, 95–98, 104].

The type strain, Nichols^T^ (=BEI Resources NR-59701^T^=DSM 117211^T^), was isolated in 1912 from the cerebrospinal fluid of a patient with neurosyphilis [67]. The genomic G+C content for the type strain is 52.8 mol% as determined by genome sequencing. The GenBank/ENA/DDBJ accession numbers for the 16S rRNA gene and the complete genome are M88726 and CP004010, respectively.

## Description of *Treponema pallidum* subspecies *pallidum* comb. nov.

*Treponema pallidum* subspecies *pallidum* comb. nov. (pal’li.dum. L. neut. adj. *pallidum*, pale, pallid)

Basonym: *Treponema pallidum* (Schaudinn and Hoffmann [14]) Schaudinn [15] (Approved Lists 1980)

*T. pallidum* subsp. *pallidum* is the cause of venereal and congenital syphilis in humans and is an obligate pathogen of humans. Cutaneous inoculation of rabbits produces skin lesions. Cutaneous inoculation of hamsters, mice and guinea pigs typically produces either no lesions or transient pathology, although long-term infection can be detected in hamsters and guinea pigs through rabbit infectivity testing. *T. pallidum* subsp. *pallidum* can be cultivated continuously in a tissue culture system [26–28, 30] or propagated by intratesticular inoculation of rabbits.

The organism is microaerophilic, and glucose is metabolized through the Embden–Meyerhof–Parnas and hexose monophosphate pathways [105]. Oxygen uptake by *T. pallidum* has been reported and is glucose-dependent [106, 107]. Oxidation of pyruvate occurs only when oxygen is present [108]. Major fermentation products of glucose are acetate and CO_2_ [109]. Requires serum for *in vitro* survival and multiplication [30, 110].

*T. pallidum* subsp. *pallidum* strains may be identified and differentiated from the other subspecies by whole-genome sequence comparisons or by the use of the SSRs described in this publication.

The type strain, Nichols^T^ (=BEI Resources NR-59701^T^=DSM 117211^T^) was isolated in 1912 from the cerebrospinal fluid of a patient with neurosyphilis [67]. The genomic G+C content for the type strain is 52.8 mol% as determined by genome sequencing. The GenBank/ENA/DDBJ accession numbers for the 16S rRNA gene and the complete genome are M88726 and CP004010, respectively. *T. pallidum* subsp. *pallidum* SS14 (=BEI Resources NR-60825=DSM 120345) serves as a reference strain representative of the so-called SS14 lineage/cluster of this subspecies.

## Description of *Treponema pallidum* subspecies *pertenue* comb. nov.

*Treponema pallidum* subspecies *pertenue* comb. nov. (per.te’nu.e. L. neut. adj. *pertenue*, extremely slight, very thin, slender.)

Basonym: *‘Spirochaeta pertenuis*’ Castellani [16]

*T. pallidum* subsp. *pertenue* is pathogenic to its natural hosts, humans and NHPs, and is not found elsewhere in nature. The organism causes yaws in humans, a contagious disease that is spread by skin-to-skin contact [2]. Naturally occurring infections in NHPs in Africa are apparently transmitted either sexually or through skin-to-skin contact [111]. Cutaneous lesions are produced at the site of inoculation in rabbits and Syrian hamsters, but not in guinea pigs. Sera from patients with yaws give positive results with serological tests for syphilis. *In vitro* culture of *T. pallidum* subsp. *pertenue* has been achieved, but the organism multiplies at a much slower rate than do the other subspecies of *T. pallidum* (J. Bosák and D. Šmajs, unpublished data; D.G. Edmondson and D. L. De Lay, unpublished data).

*T. pallidum* subsp. *pertenue* can be isolated from lesions of human yaws cases or from NHP infections. The organism is present in tropical areas of Africa, Southeast Asia and the Western Pacific Islands. Yaws infections were previously present in South and Central America but have not been reported in these areas in decades.

*T. pallidum* subsp. *pertenue* strains may be identified and differentiated from the other subspecies by whole-genome sequence comparisons or by the use of the SSRs described in this publication.

The type strain, Gauthier^T^ (=BEI Resources NR-60826^T^=DSM 120346^T^), was isolated by Gastinel *et al.* in 1950 [69] through the inoculation of a hamster with skin lesion exudate from a human patient with yaws in the current Democratic Republic of the Congo. The type strain has a genomic G+C content of 52.8 mol%, as determined by genome sequencing. The GenBank/ENA/DDBJ accession number for the complete genome is CP002376.

## Description of *Treponema pallidum* subspecies *endemicum* subsp. nov.

*Treponema pallidum* subspecies *endemicum* subsp. nov. (en.de’mi.cum. Gr. masc. adj. *endemos*, native, dwelling in place. N.L. neut. adj. *endemicum*, endemic)

*T. pallidum* subsp. *endemicum* is an obligate pathogen of humans, causing bejel (also called endemic syphilis), a contagious disease typically spread in pre-pubertal years by contact with infected individuals or the shared use of contaminated utensils. Cases of *T. pallidum* subsp. *endemicum* infection that were transmitted sexually have been identified in Cuba, Japan and Pakistan, as determined by MLST or genome sequence analysis [62, 85, 86, 112]. Sera from patients with bejel give positive results with serological tests for syphilis.

*T. pallidum* subsp. *endemicum* strains have been successfully cultivated in a tissue culture system [26, 30] and can also be propagated by intratesticular inoculation of rabbits or by intradermal inoculation of hamsters. The organisms can be isolated from inguinal lymph nodes 3–4 weeks after intradermal infection. Inbred hamsters (e.g. LSH/Ss LAK) are particularly useful for study of this organism [113]. Produces lesions in rabbits, hamsters and guinea pigs but not in mice. *T. pallidum* subsp. *endemicum* strains may be identified and differentiated from the other subspecies by whole-genome sequence comparisons or by the use of the SSRs described in this publication.

The type strain, Bosnia A^T^ (=BEI Resources NR-60824^T^=DSM 120347^T^) was obtained by E. I. Grin in 1950 using the inoculation of hamsters with exudate from an oral lesion from a patient with bejel, as later described by Turner and Hollander [70]. The genomic G+C content of the type strain is 52.8 mol%, as determined by genome sequencing. The GenBank/ENA/DDBJ accession number for the complete genome is CP007548.

## Supplementary material

10.1099/ijsem.0.007245 Supplementary Material 1.

10.1099/ijsem.0.007245 Supplementary Material 2.

10.1099/ijsem.0.007245 Supplementary Material 3.

## Abbreviations

CDC: Centers for Disease Control and Prevention
DSMZ: Deutsche Sammlung von Mikroorganismen und Zellkulturen
ENA: European Nucleotide Archive
MLST: multi-locus sequence typing
NCBI: National Center for Biomedical Informatics
NHPs: non-human primates
SNV: single nucleotide variation
SSR: subspecies-specific region
