# Supplementary material for: Formal description of Treponema pallidum subsp. pallidum, comb. nov., Treponema pallidum subsp. pertenue, comb. nov., Treponema pallidum subsp. endemicum, subsp. nov. and emended description of Treponema pallidum
Source: Int J Syst Evol Microbiol. 2026 Jul 23;76(7):007245. doi: 10.1099/ijsem.0.007245 (PMC13398306; doi:10.1099/ijsem.0.007245)
Supplement: Supplementary Material 1. [file ijsem-76-07245-s001.pdf]

Supplemental Materials

Formal description of *Treponema pallidum* subsp. *pallidum*, comb. nov., *Treponema pallidum* subsp. *pertenue*, subsp. nov., *Treponema pallidum* subsp. *endemicum*, subsp. nov., and emended description of *Treponema pallidum*

Steven J. Norris, Diane G. Edmondson, Bridget D. De Lay, Karan G. Kaval, Tsute Chen, Nicole A. P. Lieberman, Alexander L. Greninger, Klára Janečková, and David Šmajs

Fig. S1. Subspecies-specific regions (SSRs) identified through comparison of representative strains of *T. pallidum* subsp. *pallidum*, subsp. *endemicum*, and subsp. *pertenue*, along with *T. paraluiscuniculi*.

Table S1. *T. pallidum* genomic sequences utilized for phylogenetic analysis in this study.

Table S2. Genome sequence differences specific for *T. pallidum* subspecies and *T. paraluiscuniculi*.

File S1 (Separate File). Analysis of *T. pallidum* sequences from a database of 3008 paired-end sequencing read sets.

Movie S1 (Separate File). Video showing the characteristic motility of *T. pallidum*, as exemplified by in vitro cultured *T. pallidum* subsp. *pallidum* Nichols^T. Darkfield microscopy was performed with a Nikon E600 Microscope using a 100X objective lens with diaphragm, a CytoViva darkfield illumination system, a PCO.Panda Camera, and Nikon Elements software. The specimen consisted of a freshly prepared *T. pallidum* suspension (~2 x 10^7 per ml) in TpCM2 medium with 1% (w/v) methyl cellulose added to increase viscosity.

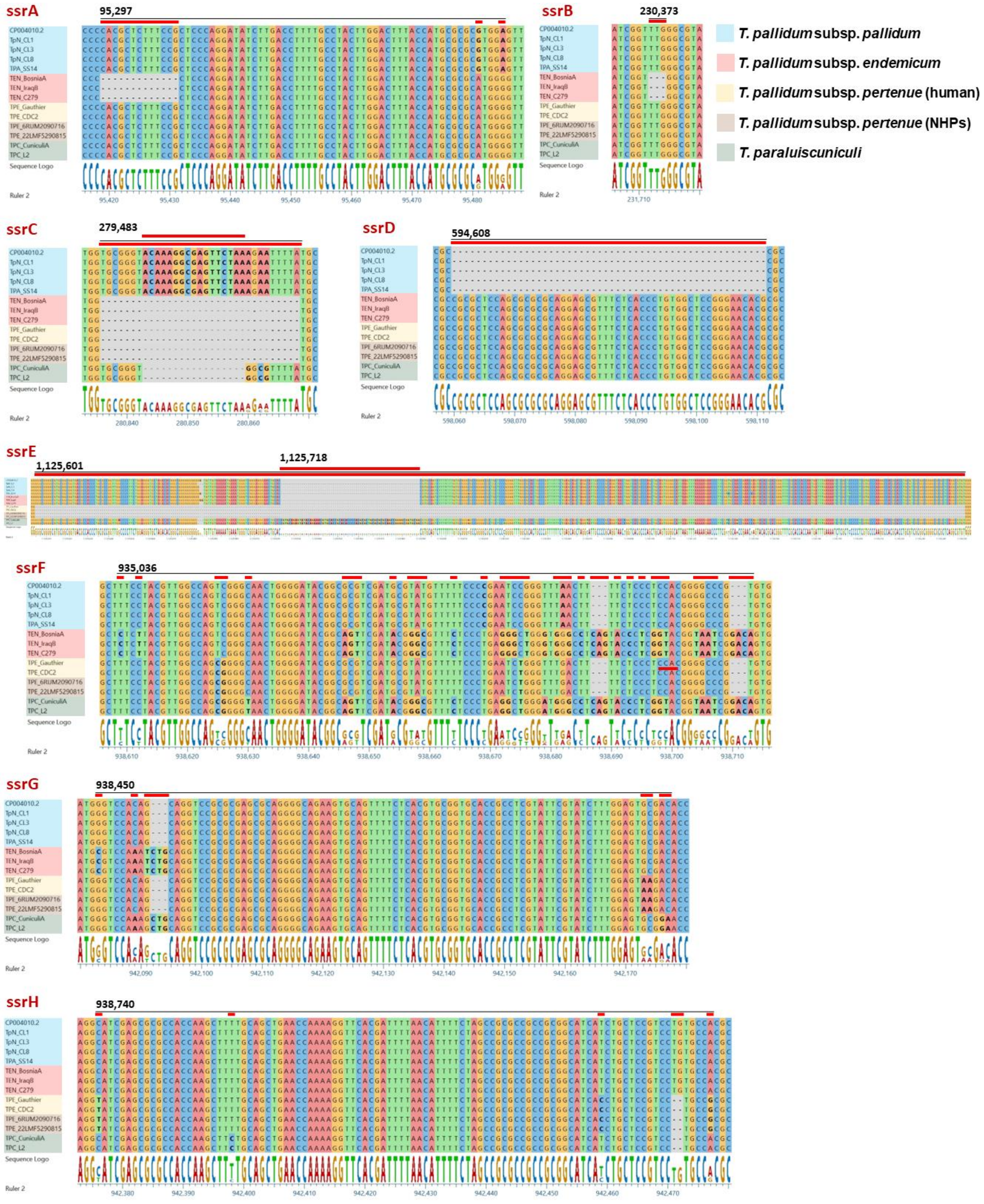

Fig. S1. Subspecies-specific regions (SSRs) identified through comparison of representative strains of *T. pallidum* subsp. *pallidum*, subsp. *endemicum*, and subsp. *pertenue*, along with *T. paraluiscuniculi*. The regions are designated ssrA through ssrH, and the beginning coordinate in the reference *T. pallidum* subsp. *pallidum* Nichols^{T} genome (CP004010.2)

is indicated for each SSR. The corresponding sequences from 10 strains and 3 *T. pallidum* subsp. *pallidum* Nichols clones (which exemplify intrastrain heterogeneities in the Nichols strain) are included; the taxa are color-coded as shown in the key. ssrE is based entirely on presence (ssrE-2) or absence (ssrE-1) of a ~379 bp relative deletion, because the sequences in the relative insert region exhibit heterogeniety (SNVs and a variable homopolymeric G sequence) within the available *T. pallidum* subsp. *pallidum* and *T. pallidum* subsp. *pertenue* genomic sequences. Additional information regarding the SSRs is provided in Table S2

**Table S1. *T. pallidum* genomic sequences utilized for phylogenetic analysis in this study.**							
**Subspecies**	**Strain**	**Specime n obtained**	**Location**	**Source[a]**	**Material**	**GenBank Accession**	**Reference(s)**
T. pallidum subsp. *pallidum*	Nichols[T]	1912	USA: Washington, DC	CSF	Isolate	CP004010.2	[1, 2]
T. pallidum subsp. *pallidum*	Chicago	1951	USA: Chicago, Illinois	Primary lesion	Isolate	CP001752.1	[3, 4]
T. pallidum subsp. *pallidum*	Haiti B	1951	Haiti: Cotes-de-Fer	"Frambesiform yaws" lesion	Isolate	CP032623.1	[3]
T. pallidum subsp. *pallidum*	Mexico A	1953	Mexico	Primary lesion	Isolate	CP003064.1	[3, 5]
T. pallidum subsp. *pallidum*	Madras	1954	India: Madras	Unknown	Isolate	CP078121.1	-- (R. George, CDC)
T. pallidum subsp. *pallidum*	SS14	1977	USA: Atlanta, Georgia	Lesion exudate	Isolate	CP004011.1	[2, 6]
T. pallidum subsp. *pallidum*	Grady	1980	USA: Atlanta, Georgia	Primary lesion	Isolate	CP035104.1	[7]
T. pallidum subsp. *pallidum*	Sea81-4	1980	USA: Seattle, Washington	Primary lesion	Isolate	CP003679.1	[8, 9]
T. pallidum subsp. *pallidum*	DAL-1	1991	USA: Dallas, Texas	Amniotic fluid, secondary syphilis during pregnancy	Isolate	CP003115.1	[10, 11]
T. pallidum subsp. *pallidum*	Amoy	2011	China: Xiamen	Primary lesion	Isolate	CP015162.1	[12]
T. pallidum subsp. *pallidum*	MD22xe	1999	USA	Swab	DNA	CP073485.2	[13]
T. pallidum subsp. *pallidum*	UAB71i	2001	Madagascar	Swab	DNA	CP073385.2	[13]
T. pallidum subsp. *pallidum*	UAB81xi	2001	Madagascar	Swab	DNA	CP073382.2	[13]
T. pallidum subsp. *pallidum*	UAB211i	2002	Madagascar	Swab	DNA	CP073447.2	[13]
T. pallidum subsp. *pallidum*	UAB515xi	2006	Madagascar	Swab	DNA	CP073417.2	[13]
T. pallidum subsp. *pallidum*	UAB528ei	2006	Madagascar	Swab	DNA	CP073410.2	[13]
T. pallidum subsp. *pallidum*	China11	2018	China	Swab	DNA	CP073574.2	[13]
T. pallidum subsp. *pallidum*	China23	2018	China	Swab	DNA	CP073573.2	[13]
T. pallidum subsp. *pallidum*	China31	2018	China	Swab	DNA	CP073571.2	[13]
T. pallidum subsp. *pallidum*	China47	2018	China	Swab	DNA	CP073568.2	[13]
T. pallidum subsp. *pallidum*	CW83	2015	Cuba	Genital smear	DNA	CP034917.1	[7]
T. pallidum subsp. *pallidum*	Dublin54B	2002	Ireland: Dublin	Swab	DNA	CP073559.2	[13]
T. pallidum subsp. *pallidum*	Dublin55B	2002	Ireland: Dublin	Swab	DNA	CP073558.2	[13]
T. pallidum subsp. *pallidum*	Dublin37B	2002	Ireland: Dublin	Swab	DNA	CP073564.2	[13]
T. pallidum subsp. *pallidum*	Dublin57B	2002	Ireland: Dublin	Swab	DNA	CP073557.2	[13]
T. pallidum subsp. *pallidum*	Italy17LGMi	2017	Italy	Swab	DNA	CP073576.2	[13]
T. pallidum subsp. *pallidum*	Italy8PLe	2017	Italy	Swab	DNA	CP073554.2	[13]
T. pallidum subsp. *pallidum*	ItalyGE4b	2017	Italy	Swab	DNA	CP073550.2	[13]
T. pallidum subsp. *pallidum*	ItalyGE5b	2017	Italy	Swab	DNA	CP073548.2	[13]
T. pallidum subsp. *pallidum*	Japan288	2019	Japan	Swab	DNA	CP073545.2	[13]
T. pallidum subsp. *pallidum*	Japan306	2019	Japan	Swab	DNA	CP073534.2	[13]
T. pallidum subsp. *pallidum*	Japan328x	2019	Japan	Swab	DNA	CP073517.2	[13]
T. pallidum subsp. *pallidum*	Japan287e	2019	Japan	Swab	DNA	CP073546.2	[13]
T. pallidum subsp. *pallidum*	Japan348	2019	Japan	Swab	DNA	CP073505.2	[13]
T. pallidum subsp. *pallidum*	Japan368i	2020	Japan	Swab	DNA	CP073491.2	[13]
T. pallidum subsp. *pallidum*	Japan338xei	2019	Japan	Swab	DNA	CP073512.2	[13]
T. pallidum subsp. *pallidum*	Japan317xi	2019	Japan	Swab	DNA	CP073526.2	[13]
T. pallidum subsp. *pallidum*	NYMC01	2021	USA: New York State	FFPE tissue	DNA	CP125219.1	[14]

T. pallidum subsp. *pallidum*	PeruC10010x	2019	Peru	Swab	DNA	CP073470.2	[13]
T. pallidum subsp. *pallidum*	Peru213041xe	2018	Peru	Swab	DNA	CP073475.2	[13]
T. pallidum subsp. *pallidum*	Peru213161xe	2018	Peru	Swab	DNA	CP073474.2	[13]
T. pallidum subsp. *pallidum*	PeruC40040x	2019	Peru	Swab	DNA	CP073467.2	[13]
T. pallidum subsp. *pallidum*	PT_SIF0697	2009	Portugal	Penile	DNA	CP016045.1	[15]
T. pallidum subsp. *pallidum*	PT_SIF1002	2011	Portugal	Penile	DNA	CP016051.1	[15]
T. pallidum subsp. *pallidum*	PT_SIF1020	2011	Portugal	Penile	DNA	CP016052.1	[15]
T. pallidum subsp. *pallidum*	PT_SIF1135	2013	Portugal	Anal	DNA	CP016055.1	[15]
T. pallidum subsp. *pallidum*	PT_SIF1140	2013	Portugal	Anal	DNA	CP016056.1	[15]
T. pallidum subsp. *endemicum*	Bosnia A[T]	1950	Bosnia	Lesion exudate	Isolate	CP007548.1	[3, 16, 17]
T. pallidum subsp. *endemicum*	Iraq B	1951	Iraq	Lesion exudate	Isolate	CP032303.1	[3, 18]
T. pallidum subsp. *endemicum*	C77	2014	Cuba: Havana	Genital ulcer exudate	DNA	CP081507.1	[19, 20]
T. pallidum subsp. *endemicum*	C279	2017	Cuba: Havana	Genital ulcer exudate	DNA	CP078090.1	[19, 20]
T. pallidum subsp. *endemicum*	Japan320e	2019	Japan	Swab	DNA	CP073523.1	[13]
T. pallidum subsp. *endemicum*	Japan322e	2019	Japan	Swab	DNA	CP073522.1	[13]
T. pallidum subsp. *endemicum*	Japan326e	2019	Japan	Swab	DNA	CP073518.1	[13]
T. pallidum subsp. *endemicum*	Japan346e	2019	Japan	Swab	DNA	CP073506.1	[13]
T. pallidum subsp. *pertenue*	SamoaD	1953	Apia, Western Samoa	Lesion exudate	Isolate	CP002374.1	[3, 21]
T. pallidum subsp. *pertenue*	Gauthier[T]	1960	Brazzaville, Congo	Lesion exudate	Isolate	CP002376.1	[21, 22]
T. pallidum subsp. *pertenue*	CDC 2575	1980	Ghana	Human skin lesion	Isolate	CP020366.1	[23]
T. pallidum subsp. *pertenue*	CDC-1	1980	Densuso, Ghana	Papillomatous lesion exudate	Isolate	CP024750.1	[24, 25]
T. pallidum subsp. *pertenue*	CDC-2	1980	Akorabo, Ghana	Lesion exudate	Isolate	CP002375.1	[21, 25]
T. pallidum subsp. *pertenue*	Ghana-051	1988	Ghana	Secondary skin lesion	Isolate	CP020365.1	[23, 26]
T. pallidum subsp. *pertenue*	Kampung Dalan K363	1990	Indonesia: Sumatra	Skin lesion	Isolate	CP024088.1	[27, 28]
T. pallidum subsp. *pertenue*	Sei Geringging K403	1990	Indonesia: Sumatra	Skin lesion	Isolate	CP024089.1	[27, 28]
T. pallidum subsp. *pertenue*	Tube7Lihir	2015	Lihir, Papua New Guinea	Swab	DNA	CP073466.1	[13]
T. pallidum subsp. *pertenue*	Fribourg-Blanc	1966	Guinea, West Africa	Lesions in *Papio papio*	Isolate	CP003902.1	[29-31]
T. pallidum subsp. *pertenue*	LMNP-1	2001	Tanzania: Lake Manyara National Park	*Papio anubis*	DNA	CP021113.1	[32]
T. pallidum subsp. *pertenue*	70M5100507	2007	Tanzania: Lake Manyara National Park	*P. anubis*	DNA	CP170149.1	[33, 34]
T. pallidum subsp. *pertenue*	09LMM2180815	2015	Tanzania: Lake Manyara National Park	*Cercopithecus mitis*	DNA	CP170143.1	[33, 35]

T. pallidum subsp. *pertenue*	19LMF8280815	2015	Tanzania: Lake Manyara National Park	*P. anubis*	DNA	CP094268.1	[24, 35]
T. pallidum subsp. *pertenue*	22LMF5290815	2015	Tanzania: Lake Manyara National Park	*P. anubis*	DNA	CP094485.1	[24, 35]
T. pallidum subsp. *pertenue*	24SNM5151115	2015	Tanzania: Serengeti National Park	*P. anubis*	DNA	CP094486.1	[24, 35]
T. pallidum subsp. *pertenue*	2SNF2130815	2015	Tanzania: Serengeti National Park	*P. anubis*	DNA	CP170144.1	[33, 35]
T. pallidum subsp. *pertenue*	6SNF2081115	2015	Tanzania: Serengeti National Park	*P. anubis*	DNA	CP170145.1	[33, 35]
T. pallidum subsp. *pertenue*	7SNM5081115	2015	Tanzania: Serengeti National Park	*P. anubis*	DNA	CP094296.1	[24, 35]
T. pallidum subsp. *pertenue*	14RUF5130716	2016	Tanzania: Ruaha National Park	*P. cynocephalus*	DNA	CP170146.1	[33, 35]
T. pallidum subsp. *pertenue*	6RUM2090716	2016	Tanzania: Ruaha National Park	*Chlorocebus pygerythrus*	DNA	CP094202.1	[24, 35]
T. pallidum subsp. *pertenue*	18NCF8220317	2017	Tanzania: Ngorongoro Conservation Area	*P. anubis*	DNA	CP094297.1	[24, 35]
T. pallidum subsp. *pertenue*	32LMM2190317	2017	Tanzania: Lake Manyara National Park	*C. pygerythrus*	DNA	CP094487.1	[24, 35]
T. pallidum subsp. *pertenue*	34LMM2190317	2017	Tanzania: Lake Manyara National Park	*C. pygerythrus*	DNA	CP094488.1	[24, 35]
T. paraluiscuniculi	Cuniculi A	1939	Unknown	Rabbit lesion	Isolate	CP002103.1	[3, 36]
T. paraluiscuniculi	V3603-13 (L2)	2013	Sweden	*Lepus timidus* skin lesion	DNA	CP097901.1	[37]

[a] All specimens are from *Homo sapiens* unless otherwise indicated. Blood, genital swab, or skin tissue biopsy were used for non-human primate specimen collection. *Cercopithecus mitis*: blue monkey. *Chlorocebus pygerythrus*: vervet monkeys. *Papio anubis*: olive baboons. *Papio cynocephalus*: yellow baboons. *Papio papio*: Guinea baboons.

Table S2. Genome sequence differences specific for *T. pallidum* subspecies and *T. paraluiscuniculi*. The reference strains shown exemplify the predominant differences seen within each taxon.[a]

					Locations and differences in each strain [b] (Differences relative to *T. pallidum* subsp. *pallidum* Nichols[T])				
**Subspecies-Specific Region (SSR)**	**Gene(s)**	**Alignment Coordinates[d]**	**Type**	**Genetic Differences, Potential Effects**	***T. pallidum* subsp. *pallidum* Nichols[T]**	***T. pallidum* subsp. *pallidum* SS14**	***T. pallidum* subsp. *endemicum* Bosnia A[T]**	***T. pallidum* subsp. *pertenue* Gauthier[T]**	***T. paraluiscuniculi* Cuniculi A**
ssrA	IGS between TP_0085 (PTS fru component IIA), TP_0086 (PilZ domain protein)	95419-95485	Indel, SNVs	Start codon variation in TP_0086 in TPA (GTG) vs. TPE and TEN (ATG); potential effect on translation efficiency	95297-95363 (--)	95297-95363 (--)	95283-95336 (13 bp deletion, 2 SNVs)	95287-95353 (2 SNVs)	95385-95451 (2 SNVs)
ssrB	TP_0225 (short-chain fatty acid importer)	231712-231714	Indel	3-bp indel resulting in 1 AA (Leu 15) deletion in TEN strains vs. TPA and TPE	230373-230375 (--)	230415-230417 (--)	230333 (3 bp deletion [TTG])	230291-230293 (--)	227796-227798 (--)
ssrC	IGS between TP_0265 (branched AA:cation symporter), TP_r0004 (16S rRNA)	280836-280868	Indel	Distinct indel patterns in the TPA, (TEN=TPE), and TPC groups	279483-279515 (--)	279525-279557 (--)	279449 (33 bp deletion)	279403 (33 bp deletion)	276882 (17 bp deletion)
ssrD	IGS between TP_0548 (FadL homolog), TP_0549 (S14 family endopeptidase ClpA)	598060-598111	Indel	52-bp deletion in region upstream of ClpA gene in TPA; potential effect on gene transcription	594608 (--)	594491 (--)	592543-592594 (52 bp insertion)	595030-595081 (52 bp insertion)	590464-590515 (52 bp insertion)
ssrE[d]	IGS between TP_1029 (DbpA RNA binding domain-containing protein homolog) and TP_1031 (TprL)	1129805-1130251	Indel, Many SNVs	TPE tprL gene has an altered, hybrid 5' end due to a 379-bp deletion, potentially affecting β-barrel architecture; SNVs within subspecies in this region	1125601-1125979 (--)	1125537-1125915 (--)	1123622-1123999 (--)	1125764 (379 bp relative deletion)	1119399-1119465 (67 bp insertion)
ssrF	TP_0856 (FadL homolog)	938609-938713	Indel, SNVs	99-105 bp region with divergence specific for each subspecies and TPC, causing differences in predicted extracellular loops of the TP_0856 protein	935036-935134 (--)	934916-935014 (--)	933013- 933117 (Multiple SNVs, 6 bp insertion)	935207- 935305 (Multiple SNVs)	928283-928387 (Multiple SNVs, 6 bp insertion)
ssrG	TP_0859 (FadL homolog)	942083-942177	Indel, SNVs	92-95 bp region with sparse SNVs and indel regions specific for each subspecies and TPC. Putative differences in outer membrane loops of TPA vs. TPE vs. TEN	938450-938541 (--)	938330-938421 (--)	936430-936524 (3 SNVs, 3 bp insertion [CTG])	938560-938651 (3 SNVs)	931741-931835 (4 SNVs, 3 bp insertion [CTG])
ssrH	TP_0859 and IGS between TP_0859 and TP_0860	942376-942477	Indel, SNVs	TPE has synonymous mutation in TP_0859, GGC-->GGT (Gly) and 2 SNVs + 2 bp deletion in IGS	938740-938841 (--)	938620-938721 (--)	936723-936824 (--)	938850-938949 (3 SNVs, 2 bp deletion)	932034-932133 (1 SNV, 2 bp deletion)

[a] Abbreviations: TPA, *T. pallidum* subsp. *pallidum*; TEN, *T. pallidum* subsp. *endemicum*; TPE, *T. pallidum* subsp. *pertenue*; TPC, *T. paraluiscuniculi*; IGS = intergenic sequence; NS = nonsynonymous; SNV = single nucleotide variation; Indel = insertion/deletion

[b] The SSR location in the genomic sequence of each strain is indicated. For relative deletions, the first bp after the deletion is provided.

[c] Coordinates correspond to those of the aligned sequences provided in File S1.

[d] TPA and TEN strains have intra-subspecies SNVs and homopolymeric G variations in ssrE, so ssrE is based entirely on the presence (ssrE-2) or absence (ssrE-1) of a ~379 bp relative deletion.

References

1. **Nichols HJ, Hough WH**. Demonstration of *Spirochaeta pallida* in the cerebrospinal fluid from a patient with nervous relapse following the use of salvarsan. *J Amer Med Assoc* 1913;60:108-110.
2. **Pětrošová H, Pospíšilová P, Strouhal M, Čejková D, Zobaníková M et al.** Resequencing of *Treponema pallidum* ssp. *pallidum* strains Nichols and SS14: correction of sequencing errors resulted in increased separation of syphilis treponeme subclusters. *PLoS One* 2013;8(9):e74319.
3. **Turner TB, Hollander DH**. *Biology of the treponematoses*. Geneva: World Health Organization; 1957.
4. **Giacani L, Jeffrey BM, Molini BJ, Le HT, Lukehart SA et al.** Complete genome sequence and annotation of the *Treponema pallidum* subsp. *pallidum* Chicago strain. *J Bacteriol* 2010;192(10):2645-2646.
5. **Pětrošová H, Zobaníková M, Čejková D, Mikalová L, Pospíšilová P et al.** Whole genome sequence of *Treponema pallidum* ssp. *pallidum*, strain Mexico A, suggests recombination between yaws and syphilis strains. *PLoS Negl Trop Dis* 2012;6(9):e1832.
6. **Stamm LV, Kerner TC, Bankaitis VA, Bassford PJ**. Identification and preliminary characterization of *Treponema pallidum* protein antigens expressed in *Escherichia coli*. *Infect Immun* 1983;41:709-721.
7. **Grillová L, Oppelt J, Mikalová L, Nováková M, Giacani L et al.** Directly sequenced genomes of contemporary strains of syphilis reveal recombination-driven diversity in genes encoding predicted surface-exposed antigens. *Front Microbiol* 2019;10:1691.
8. **Giacani L, Iverson-Cabral SL, King JC, Molini BJ, Lukehart SA et al.** Complete genome sequence of the *Treponema pallidum* subsp. *pallidum* Sea81-4 strain. *Genome Announc* 2014;2(2).
9. **Giacani L, Chattopadhyay S, Centurion-Lara A, Jeffrey BM, Le HT et al.** Footprint of positive selection in *Treponema pallidum* subsp. *pallidum* genome sequences suggests adaptive microevolution of the syphilis pathogen. *PLoS Negl Trop Dis* 2012;6(6):e1698.
10. **Zobaníková M, Mikolká P, Čejková D, Pospíšilová P, Chen L et al.** Complete genome sequence of *Treponema pallidum* strain DAL-1. *Stand Genomic Sci* 2012;7(1):12-21.
11. **Wendel GD, Jr., Sanchez PJ, Peters MT, Harstad TW, Potter LL et al.** Identification of *Treponema pallidum* in amniotic fluid and fetal blood from pregnancies complicated by congenital syphilis. *Obstet Gynecol* 1991;78(5 Pt 2):890-895.
12. **Tong ML, Zhao Q, Liu LL, Zhu XZ, Gao K et al.** Whole genome sequence of the *Treponema pallidum* subsp. *pallidum* strain Amoy: An Asian isolate highly similar to SS14. *PLoS One* 2017;12(8):e0182768.
13. **Lieberman NAP, Lin MJ, Xie H, Shrestha L, Nguyen T et al.** *Treponema pallidum* genome sequencing from six continents reveals variability in vaccine candidate genes and dominance of Nichols clade strains in Madagascar. *PLoS Negl Trop Dis* 2021;15(12):e0010063.
14. **Velasquez MR, De Lay BD, Edmondson DG, Wormser GP, Norris SJ et al.** A novel *Treponema pallidum* subspecies *pallidum* strain associated with a painful oral lesion Is a member of a potentially emerging Nichols-related subgroup. *Sex Transm Dis* 2024;51(7):486-492.
15. **Pinto M, Borges V, Antelo M, Pinheiro M, Nunes A et al.** Genome-scale analysis of the non-cultivable Treponema pallidum reveals extensive within-patient genetic variation. *Nat Microbiol* 2016;2:16190.
16. **Turner TB, Hollander DH**. Studies on treponemes from cases of endemic syphilis. *Bull World Health Organ* 1952;7:75-81.
17. **Štaudová B, Strouhal M, Zobaníková M, Čejková D, Fulton LL et al.** Whole genome sequence of the *Treponema pallidum* subsp. *endemicum* strain Bosnia A: the genome is related to yaws treponemes but contains few loci similar to syphilis treponemes. *PLoS Negl Trop Dis* 2014;8(11):e3261.
18. **Mikalová L, Janečková K, Nováková M, Strouhal M, Čejková D et al.** Whole genome sequence of the Treponema pallidum subsp. endemicum strain Iraq B: A subpopulation of bejel treponemes contains full-length tprF and tprG genes similar to those present in T. p. subsp. pertenue strains. *PLoS One* 2020;15(4):e0230926.
19. **Vrbová E, Noda AA, Grillová L, Rodriguez I, Forsyth A et al.** Whole genome sequences of *Treponema pallidum* subsp. *endemicum* isolated from Cuban patients: The non-clonal character of isolates suggests a persistent human infection rather than a single outbreak. *PLoS Negl Trop Dis* 2022;16(6):e0009900.
20. **Noda AA, Grillová L, Lienhard R, Blanco O, Rodriguez I et al.** Bejel in Cuba: molecular identification of *Treponema pallidum* subsp. *endemicum* in patients diagnosed with venereal syphilis. *Clin Microbiol Infect* 2018.

21. **Čejková D, Zobaníková M, Chen L, Pospíšilová P, Strouhal M et al.** Whole genome sequences of three *Treponema pallidum* ssp. *pertenue* strains: yaws and syphilis treponemes differ in less than 0.2% of the genome sequence. *PLoS Negl Trop Dis* 2012;6(1):e1471.
22. **Gastinel P, Vaisman A, Hamelin A, Dunoyer F**. [Study of a recently isolated strain of *Treponema pertenue*]. *Ann Dermatol Syphiligr (Paris)* 1963;90:155-161.
23. **Strouhal M, Mikalová L, Havlíčková P, Tenti P, Čejková D et al.** Complete genome sequences of two strains of *Treponema pallidum* subsp. *pertenue* from Ghana, Africa: Identical genome sequences in samples isolated more than 7 years apart. *PLoS Negl Trop Dis* 2017;11(9):e0005894.
24. **Janečková K, Roos C, Fedrová P, Tom N, Čejková D et al.** The genomes of the yaws bacterium, *Treponema pallidum* subsp. *pertenue*, of nonhuman primate and human origin are not genomically distinct. *PLoS Negl Trop Dis* 2023;17(9):e0011602.
25. **Liska SL, Perine PL, Hunter EF, Crawford JA, Feeley JC**. Isolation and transportation of *Treponema pertenue* in golden hamsters. *Curr Microbiol* 1982;7:41-43.
26. **Engelkens HJ, Oranje AP, Stolz E**. Early yaws, imported in The Netherlands. *Genitourin Med* 1989;65(5):316-318.
27. **Noordhoek GT, Engelkens HJH, Judanarso J, van der Stek J, Aelbers GNM et al.** Yaws in West Sumatra, Indonesia: Clinical manifestations, serological findings, and characterisation of new *Treponema* isolates by DNA probes. *Eur J Clin Microbiol Infect Dis* 1991;10:12-19.
28. **Strouhal M, Mikalová L, Haviernik J, Knauf S, Bruisten S et al.** Complete genome sequences of two strains of *Treponema pallidum* subsp. *pertenue* from Indonesia: Momarrdular structure of several treponemal genes. *PLoS Negl Trop Dis* 2018;12(10):e0006867.
29. **Fribourg-Blanc A, Mollaret HH, Niel G**. [Serologic and microscopic confirmation of treponemosis in Guinea baboons]. *Bull Soc Pathol Exot Filiales* 1966;59(1):54-59.
30. **Fribourg-Blanc A, Mollaret HH**. Natural treponematosis of the African primate. *Primates Med* 1969;3(0):113-121.
31. **Zobaníková M, Strouhal M, Mikalová L, Čejková D, L A et al.** Whole genome sequence of the *Treponema* Fribourg-Blanc: unspecified simian isolate is highly similar to the yaws subspecies. *PLoS Negl Trop Dis* 2013;7(4):e2172.
32. **Knauf S, Gogarten JF, Schuenemann VJ, De Nys HM, Dux A et al.** Nonhuman primates across sub-Saharan Africa are infected with the yaws bacterium *Treponema pallidum* subsp. *pertenue*. *Emerg Microbes Infect* 2018;7(1):157.
33. **Janečková K, Roos C, Andrla P, Fedrová P, Tom N et al.** Whole-genome sequencing reveals evidence for inter-species transmission of the yaws bacterium among nonhuman primates in Tanzania. *PLoS Negl Trop Dis* 2025;19(2):e0012887.
34. **Knauf S, Batamuzi EK, Mlengeya T, Kilewo M, Lejora IA et al.** *Treponema* infection associated with genital ulceration in wild baboons. *Vet Pathol* 2012;49(2):292-303.
35. **Chuma IS, Batamuzi EK, Collins DA, Fyumagwa RD, Hallmaier-Wacker LK et al.** Widespread *Treponema pallidum* infection in nonhuman primates, Tanzania. *Emerg Infect Dis* 2018;24(6):1002-1009.
36. **Šmajs D, Zobaníková M, Strouhal M, Čejková D, Dugan-Rocha S et al.** Complete genome sequence of *Treponema paraluiscuniculi*, strain Cuniculi A: the loss of infectivity to humans is associated with genome decay. *PLoS One* 2011;6(5):e20415.
37. **Pospíšilová P, Čejková D, Buršíková P, Fedrová P, Mikalová L et al.** The hare syphilis agent is related to, but distinct from, the treponeme causing rabbit syphilis. *PLoS One* 2024;19(8):e0307196.
